# Cures of Animals

**Published:** 1882-07

**Authors:** C. F. Nichols

**Affiliations:** Boston


### CURES OF ANIMALS.

*Two Cases of "Spring-Halt."*

C. F. NICHOLS, M. D., BOSTON.

In the spring of 1880 my horse had been for six months pronounced worthless and incurable; the horse, a roan, weight, 900, active in old age (24 years), hitherto a remarkably tough and useful animal.

In this case the (horse) doctors did *not* disagree. The patient's hind-legs had gradually learned to twitch worse and worse, until the spasm jerked them alternately up to the belly of the animal. As usual, aggravation took place after standing in stall, worse in damp weather, a little better after prolonged exercise. Legs swelled after standing, also if strained by a long drive, when, though sensitive to touch, the swelling went down after rubbing.

Given $Rhus^{cm}$, a dry powder, every 24 hours ineffectually. Next month, learning that the horse had suffered from thrush, *with a narrow fistulous opening, running well up through the hoof*, for which he had been treated by injecting carbolic acid, I gave $Silicea^{cm}$, three doses, dry, 48 hours apart. (Find in Bœnninghausen's Pocket Book these same aggravations above noted, together with fistula.) Rapid improvement took place. The horse has been driven ever since, pronounced sound.*

With Magnes. phos. 12 cent. I have relieved another horse. This horse's *Gressus gallinaceus* recurred occasionally until he got a dose of Sulphur$^{dm}$, (S.) (given three months ago).

* It is well known among horsemen that a case of developed spring-halt is incurable and not to be palliated in any degree by treatment external or internal, rest, exercise, feed or place of standing.

